# Semicircular Patch-Embedded Vivaldi Antenna for Miniaturized UWB Radar Sensors

**DOI:** 10.3390/s20215988

**Published:** 2020-10-22

**Authors:** Jungwoo Seo, Jae Hee Kim, Jungsuek Oh

**Affiliations:** 1 Institute of New Media and Communications, Seoul National University, Seoul 08826, Korea; jw.seo@snu.ac.kr; 2 Department of Electrical and Computer Engineering, Seoul National University, Seoul 08826, Korea; 3 School of Electrical, Electronics and Communication Engineering, Korea University of Technology and Education, Cheonan 31253, Korea; jaehee@koreatech.ac.kr

**Keywords:** miniaturization methods, ultrawideband antennas, Vivaldi antennas

## Abstract

A microstrip-to-slot line-fed miniaturized Vivaldi antenna using semicircular patch embedment is proposed in this study. The conventional Vivaldi antenna has ultrawide bandwidth, but suffers from low gain in the low-frequency band. The proposed antenna topology incorporates the embedment of semicircular patch elements into the side edge of the antenna. This enables the phases of electric fields at both ends of the antenna to be out of phase. Since the distance between the two ends are λ_L_/2 where λ_L_ is the wavelength at a low operating frequency, this antenna topology can achieve the constructive addition of electrical fields at the radiating end, leading to gain enhancement at the chosen low frequency. In comparison with the conventional Vivaldi antenna, the proposed antenna has a wider bandwidth from 2.84 to 9.83 GHz. Moreover, the simulated result shows a gain enhancement of 5 dB at low frequency. This cannot be realized by the conventional low-band impedance matching techniques only relying on slotted topologies. The measured results of this proposed antenna with a size of 45 × 40 × 0.8 mm^3^ are in good agreement with the simulated results.

## 1. Introduction

The FCC (Federal Communication Commission) defines UWB (Ultra-Wide-Band) as “Wireless transmission technology with bandwidth over 20% of center frequency or over 500 MHz”. UWB is defined as a short-range wireless connectivity technology that realizes high-speed communication and radar sensing, faster than 100 Mbps, at low power over an ultra-wideband of 3.1 to 10.6 GHz. UWB systems are used in various fields. There are some applications such as the wall estimation technique by the UWB radar system [1], Vivaldi antenna for Radio Frequency (RF) energy harvesting [2], and mobbing target tracking by UWB radar [3]. One of the most widely used UWB antennas in such systems is the Vivaldi antenna, invented by Gibson in 1979 [4], which offers the advantages of a low profile, high-speed data rate, compact size, simple structure, ultra-wideband, and low cost.

In the Vivaldi antenna, the feed transition structure decides a high operating frequency band, while aperture width determines its lowest operating frequency band. In order to miniaturize the size of the Vivaldi antenna and extend its bandwidth, various methods such as the corrugated Vivaldi antenna [5], elliptically shaped arm Vivaldi antenna [6], defected ground slot [7], and tapered slot edge [8] have been proposed. Additionally, some structures are placed in front of antenna such as parasitic element [9], elliptical cylinder [10], or two-stub-loaded split-ring resonator [7] to enhance antenna gain.

Because of the structural limits in integrating the feed transition and aperture of the Vivaldi antenna into systems, many small UWB radar sensors require further size miniaturization of the Vivaldi antenna and its ground and minimizing gain degradation. First, to alleviate the structural limits, the antipodal Vivaldi antenna suggested by Gazit [11] also referred to as a symmetric double-sided slot line was constructed with the microstrip feed as an input feed. With the assistance of this transition structure, the antipodal Vivaldi antenna obtained high performances [6]. Second, to solve the gain degradation problem at the expense of antenna miniaturization, metamaterials [12] are integrated with the Vivaldi antenna. However, the aforementioned technique improves antenna gain only at high frequency bands, while contributing to the increase in the overall size of the antenna and complicating the fabrication process. Therefore, an alternative method is necessary for antennas that do not have enough space for metamaterials methods.

In such miniaturized UWB antennas, the gain variation may be too large depending on the operating frequency. For any fixed physical dimension of the antenna, the gain increases with frequency due to the enhanced radiating aperture. For reliable and robust wireless communication across the operating frequencies, it is desired that antenna gain in the lower frequency is boosted to reduce gain variation without increasing antenna size.

In this study, we propose a novel miniaturized semicircular patch embedded Vivaldi antenna (SPEVA) for gain enhancement. This structure enables expanding impedance matching toward the lower frequency band while significantly enhancing antenna gain without increasing the size. The performance of the low boundary is critical in the UWB radar sensor system. The novelty of the proposed antenna focuses on gain improvement in the low frequency band of the miniaturized UWB antennas while maintaining the gain performance in the middle and high frequency bands. The embedded semicircular patch elements render ideal standing waves of electric field at the location λ/2 away from the front side of the antenna in the low operating frequencies without losing impedance matching characteristics in the other frequency bands. This reinforces antenna gain along the main beam direction in the low operating frequencies while maintaining the overall level of antenna gain in the other frequency bands. Section 2 shows the design process, configuration such as feed method, overall structure and principle of SPEVA. Finally, SPEVA is fabricated and measured to verify the proposed design in Section 3.

## 2. Antenna Design 

### 2.1. Antenna Geometry

**Figure 1:**
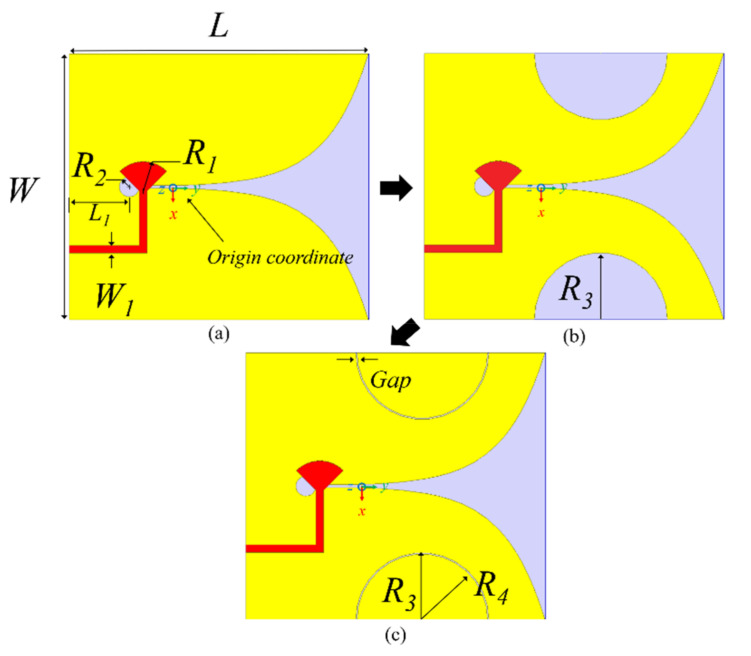
Evolution of the proposed configuration: (**a**) conventional Vivaldi antenna; (**b**) slotted Vivaldi antenna; and (**c**) semicircular patch embedded Vivaldi antenna (SPEVA).

In this study, the conventional Vivaldi antenna shown in Figure 1 is considered a reference for the initial slotted Vivaldi antenna and the proposed SPEVA. Figure 1 shows the design procedure of three Vivaldi antennas, namely, (1) a conventional Vivaldi antenna, (2) a slotted Vivaldi antenna, and (3) a SPEVA. The three antennas were designed to have the same size, and their physical dimension parameters are listed in Table 1. The overall dimension of the Vivaldi antenna is 40 × 45 × 0.8 mm^3^, which approximately corresponds to 0.4λ _L_ × 0.45λ _L_ × 0.08λ _L_, where λ _L_ is the wavelength in the lowest operating frequency. The substrate was chosen to be FR-4 with the permittivity of 5.2 and loss tangent of 0.038, which is a cost-effective and commercial substrate commonly employed for industrial mass production. The FR-4-based conventional Vivaldi antenna was used as the basic structure because the unit cost of the UWB sensor is important and effective for inexpensive substrates and commercialization. The objective of SPEVA effectively reduces manufacturing costs through simple structural modifications from conventional Vivaldi antenna. The microstrip-to-slot line transition was utilized to feed the proposed antenna. The exponential profile curves applied in this design are described by the Equation (1):

(1) $$y=\;\pm \left(0.2e^{162x}\right)$$

where: 0 mm ≤ x ≤ 20 mm 0 mm ≤ y ≤ 35 mm.

The primary modified structure of the Vivaldi antenna is shown in Figure 1b. Similar to the corrugated Vivaldi antenna [5], semicircular slots are applied for the edge of the proposed antenna for miniaturization and improving radiation performance. To excite λ/2 spaced two electric dipoles, the proposed embedded semicircular patch elements were optimized to have the electric fields act in opposite directions at both ends of the antenna. This can reinforce gain to the end-fire direction for the λ/2 spaced two effective dipoles aligned in an opposite direction against each other. Although a well known slotted Vivaldi antenna works for expanding impedance matching toward lower band, it has a critical limit in realizing the simultaneous gain enhancement in the entire frequency bands. Therefore, a new design methodology that can utilize the whole structure of the slotted Vivaldi antenna dimension further efficiently by modifying ground shape, was devised. As shown in Figure 1c, a simple topology of the proposed SPEVA having the embedded semicircular patch elements can achieve the aforementioned low-band radiation features without increasing the entire antenna dimension.

### 2.2. Antenna Analysis

**Figure 2:**
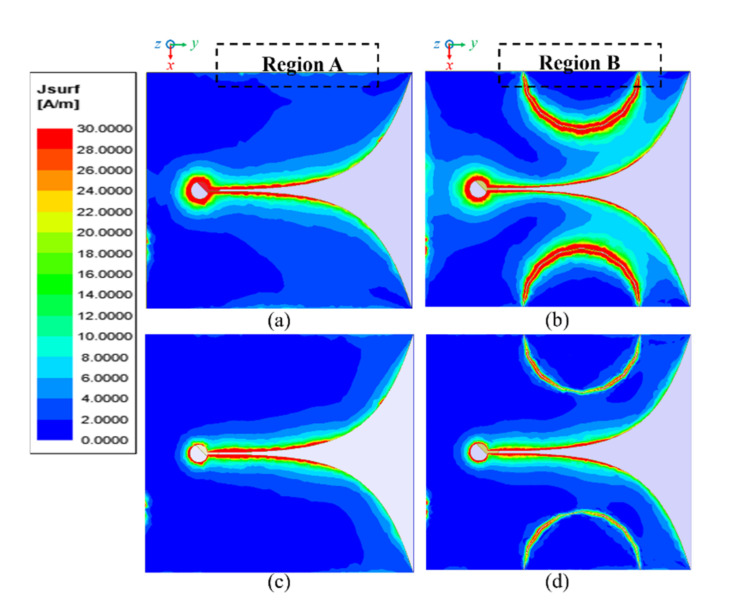
Electric surface current distributions of the conventional Vivaldi antenna and SPEVA, respectively, (**a**) and (**b**) at 3 GHz, and (**c**) and (**d**) at 7 GHz.

Figure 2 shows field distributions of the electric surface current of the conventional Vivaldi antenna and SPEVA at 3 and 7 GHz. By comparing the field distributions in the frequencies of 3 and 7 GHz, it was pre-examined that the SPEVA structure has a significant effect on low-frequency operation, but not much on the other frequencies. In Figure 2a, the electric surface current at the side edge of the antenna is dominant along region A, which is a common feature in the conventional miniaturized Vivaldi antenna having a finite ground plane. By using semicircular patch embedment, the field distributions are changed to minimize the surface currents that contribute to unnecessary radiation in the –*x* axis direction as shown in region B of Figure 2b. The excited surface currents in the slots between the ground of the Vivaldi antenna and the embedded semicircular patch generates possibilities in designing radiating fields along the side edge of the proposed SPEVA. On the other hand, reduced electric surface currents in Figure 2c,d indicate that the SPEVA structure does not degrade efficient radiation at high frequencies.

**Figure 3:**
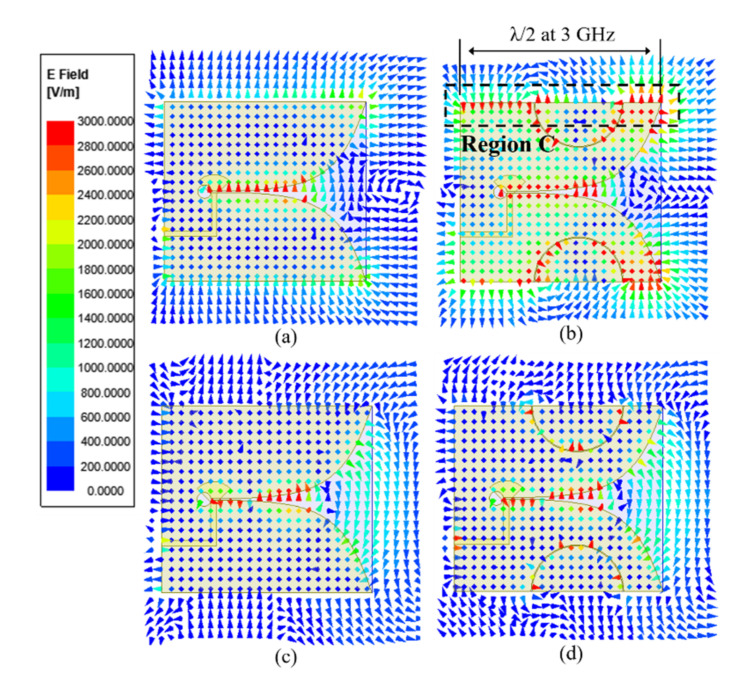
E-field distributions of the conventional Vivaldi antenna and SPEVA at (**a**,**b**) 3 GHz and (**c**,**d**) 7 GHz.

The simulated vector electric fields are presented in Figure 3 to observe the effects of the SPEVA structure. As can be observed in Figure 3a,b, as the current path becomes longer due to the semicircular patch structure, the phase direction of the E-field at both ends of region C exhibits the opposite direction. The distance between both ends of region C is λ/2 rendering a 180° phase difference at 3 GHz, which boosts radiation performance at the low frequency. As discussed earlier, the SPEVA structure does not affect radiation performance at the high frequency as shown in Figure 3c,d. In other words, if the semicircular patch is embedded in the antenna, the current distribution phase direction of both ends of the antenna is changed in reverse. The length (L) of the antenna corresponds to λ/2 at 3 GHz, improving the radiation performance in the end-fire direction by the antenna theory. Additionally, since the antenna size is larger than the high frequency wavelength, it is not affected. These behaviors contribute to improvements in bandwidth and gain without affecting high frequency performance. By employing a simple antenna topology, the aforementioned design goal is achieved for miniaturized Vivaldi antennas having a finite ground plane without increasing antenna size. 

## 3. Results and Discussion

**Figure 4:**
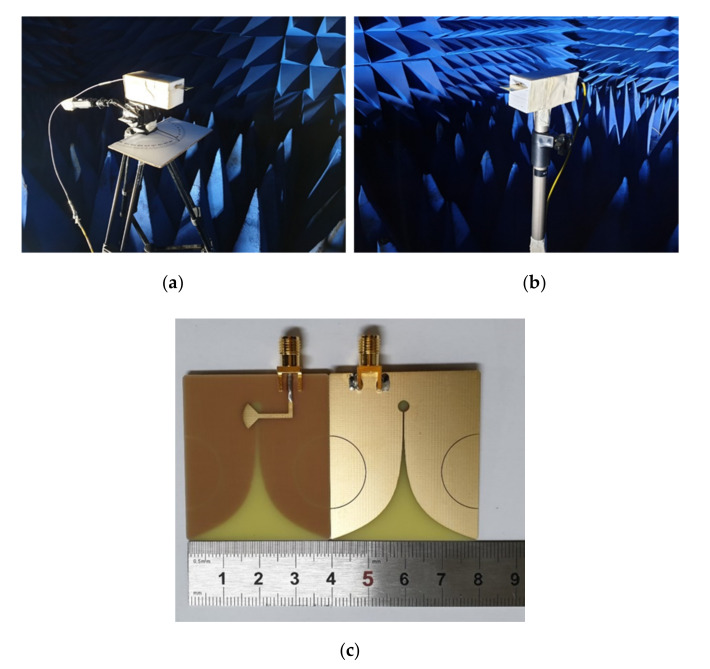
Photographs of (**a**) the Tx antenna and (**b**) the Rx antenna in an anechoic chamber and (**c**) the fabricated SPEVA.

The configurations proposed in Section 2 are simulated and optimized by ANSYS HFSS 2019 R2. By parametric optimization, the final design parameters are obtained and listed in Table 1. As shown in Figure 4c, SPEVA is fabricated and fed with a 50 Ω SubMiniature version A (SMA) connector, and finally measured in the anechoic chamber setup filmed in Figure 4a,b. Additionally, the conventional Vivaldi antennas and slotted Vivaldi antennas are made for comparison with the proposed SPEVA.

**Figure 5:**
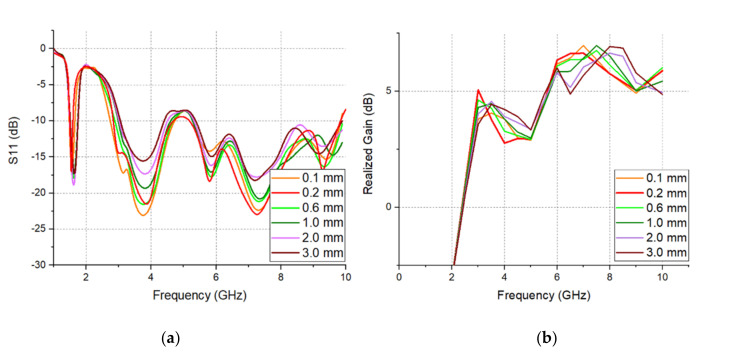
Simulated (**a**) S_11_ and (**b**) realized gain of SPEVA with the different value of Gap.

**Figure 6:**
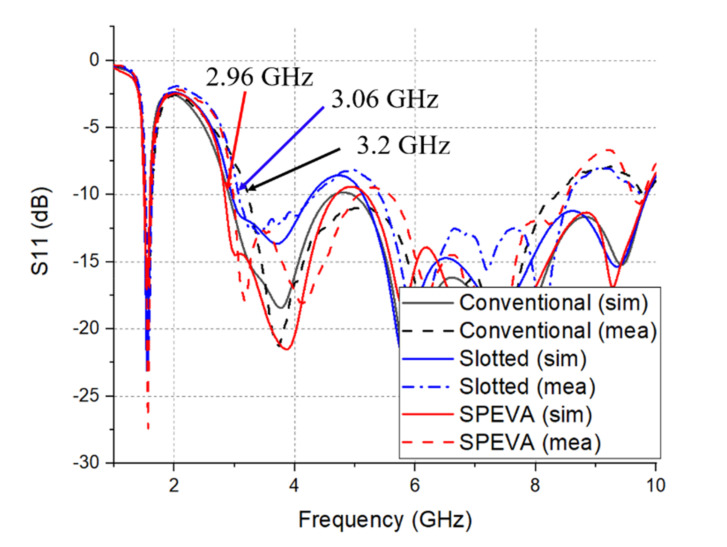
Simulated and measured S_11_ of three types of Vivaldi antennas.

The effect of the Gap parameter on the reflection coefficient and realization gain of the proposed SPEVA is simulated and studied in Figure 5. It can be observed that the interaction between the semicircular patch and the antenna increases as the gap of the semicircular patch decreases. As the gap decreases, the bandwidth becomes wider and the gain of the low-band boundary increases. Then, exceeding a certain point will degrade performance. The optimized point is 0.2 mm. Figure 6 shows variation features in S11 of the conventional Vivaldi antenna, semicircular slotted Vivaldi antenna and SPEVA, respectively. The 10-dB return loss bandwidth of the conventional Vivaldi antenna starts from 3.2 GHz, while the slotted Vivaldi antenna lowers it to 3.06 GHz. It should be noted that although the slotted Vivaldi antenna can be miniaturized, the bandwidth is decreased. The SPEVA lowers the limitation down to 2.96 GHz leading to antenna miniaturization.

**Figure 7:**
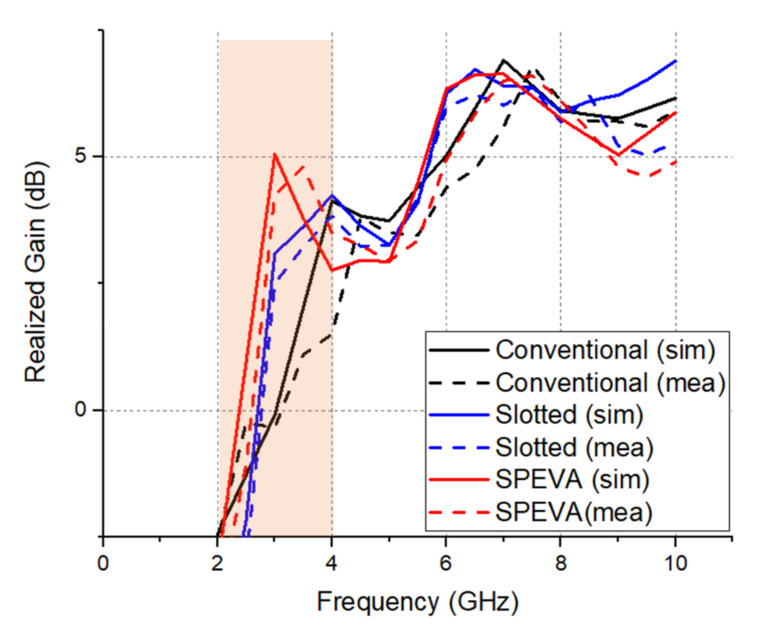
Simulated and measured realized gain of the three types of Vivaldi antennas. Gain enhancement range is highlighted.

The measured results agree well with the simulated results except for slight frequency shifts due to unstable soldering and attachment of the SMA connector. The proposed SPEVA has an ultrawide band of 2.96–5.05/5.58–8.52 GHz. Figure 7 shows simulated and measured results of the gain variations in the conventional Vivaldi antenna and SPEVA depending on the operating frequency.

**Figure 8:**
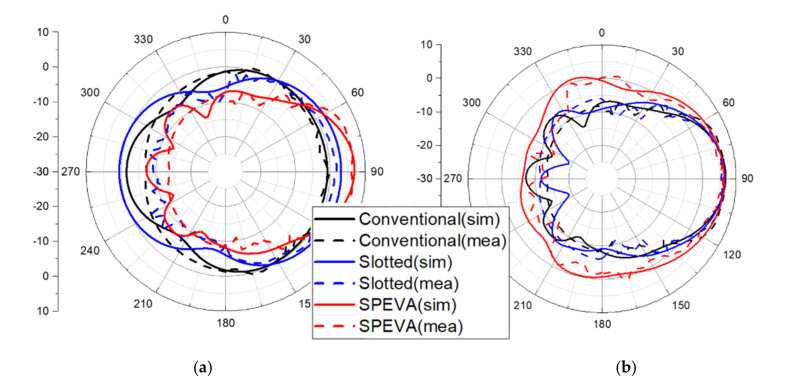
Simulated and measured radiation patterns of three types of Vivaldi antennas (**a**) at 3 GHz and (**b**) 7 GHz.

The gain of the conventional Vivaldi antenna is about 0 dBi at 3 GHz, while the gain of SPEVA is about 5 dBi. Simulated and measured results just show a difference of about 1 dB. Figure 8 shows the simulated and measured radiation patterns of the conventional slotted Vivaldi antenna and SPEVA at 3 and 7 GHz. The main lobes of the radiation patterns head to the end-fire direction as expected. Compared to conventional or slotted Vivaldi antennas, SPEVA achieves a significant gain improvement in the low frequency band. This maintains the performance in the high frequency band, solves the gain deterioration in the low frequency band of the UWB antenna mentioned above, and reduces the gain difference between the frequencies. There is a 1 dB reduction in the 4 GHz band, but the performance at 3 GHz has a significant gain improvement of 5 dB.

## 4. Conclusions

In this study, a novel miniaturized semicircular patch embedded Vivaldi antenna with a finite ground plane was presented. The semicircular slotted Vivaldi topology, while designed to change the phase of both ends of the antenna for gain enhancement at low frequencies, degrades impedance matching to the feed line hampering UWB operation. To solve the bandwidth degradation problem, the semicircular patch was embedded to the slot without increasing in antenna size. The SPEVA presents advantages of simple topologies and design parameters to improve gain at the low frequency band. This advantage makes the proposed antenna a good candidate for use in the ultrawide band and other wireless communication systems requiring miniaturized configurations.

## Figures and Tables

**Table 1:** Design parameter and their values of the proposed three antennas. All physical dimensions are in mm.

Dimension	Value	Dimension	Value
L	45	R_2_	0.75
W	40	R_3_	10
L_1_	8.25	R_4_	9.8
W_1_	1.2	Gap	0.2
R_1_	3		
